# Integrative Bioinformatics Analysis of mRNA Expression Profiles of Mice to Explore the Key Genes Involved in Crim1 Mutation-Induced Congenital Cataracts

**DOI:** 10.1007/s10528-022-10323-3

**Published:** 2022-12-31

**Authors:** Ziran Zhang, Fanke Meng, Jing Zhou, Zhihan Zhang, Xiaotian Liang, Meijun Meng, Guoguo Yi, Min Fu

**Affiliations:** 1 The Second Clinical Medicine School, Southern Medical University, Guangzhou, Guangdong China; 2 Emergency Department, Zhujiang Hospital of Southern Medical University, Industrial Avenue, Haizhu District, No. 253, Guangzhou, 510280 Guangdong China; 3 Sixth Affiliated Hospital of Sun Yat-Sen University, Yuancun, Tianhe District, 26 Er Heng Road, Guangzhou, 510655 Guangdong China; 4 ZhuJiang Hospital, Southern Medical University, Industrial Avenue, Haizhu District, No. 253, Guangzhou, 510280 Guangdong China

**Keywords:** Crim1, Congenital cataract, Differentially expressed genes, Bioinformatic analysis

## Abstract

Crim1 has been implicated in cataracts in mice and is of great importance in the development of the eye in both humans and mice. Therefore, we aimed to clarify how Crim1 mutations affect lens development and the molecular mechanism of cataracts in mice through comprehensive bioinformatics analysis. The microarray chip was downloaded from the GEO database to obtain the gene expression profile data set. Differentially expressed genes (DEGs) were screened using the limma package. GO and KEGG analyses of DEGs were performed using the DAVID database. Then, we established the protein–protein interaction (PPI) network in Cytoscape. Next, we used MCODE to analyze the data. We obtained 750 DEGs in total, including 407 upregulated DEGs and 343 downregulated DEGs. GO analysis showed that the DEGs were mainly related to biological processes, such as apoptosis, cell translation and the immune system. KEGG analysis showed that the enriched functions and pathways were related to the processing and presentation of ribosomes, lysosomes, and antigens. We identified 18 HUB genes, among which four core genes, C1qa, C1qb, C1qc, and Cd74, were closely related to congenital cataracts induced by Crim1 mutation. This study reveals the molecular pathogenesis of congenital cataracts induced by Crim1, and this information is expected to facilitate clinical genetic testing, molecular diagnosis, prognosis, and individualized chemotherapy for congenital cataracts (CC).

**Supplementary Information:**

The online version contains supplementary material available at 10.1007/s10528-022-10323-3.

## Introduction

Abnormal metabolism of the lens and decreased transparency are involved in congenital cataracts, which can cause blindness in severe cases. Approximately 1/4 of cases of congenital cataracts are caused by genetic defects (Wang et al 2016). Therefore, screening and identification of mutated genes that cause congenital cataracts can help us to understand the pathogenesis of congenital cataracts. At present, congenital cataracts are associated with greater than 200 gene mutations (Liu et al. 2018), including mutations in the crystallin gene and cytoskeleton protein gene (Zhou et al. 2018). With the development of molecular genetics, especially gene technology, an increasing number of gene mutations have been demonstrated to be related to the pathogenesis of congenital cataracts. Studies have shown that mutation of any gene involved in lens development may lead to congenital cataract (Shiels et al. 2007). In addition, acetylation of crystallin is also related to the occurrence of congenital cataract. However, the occurrence and development of CC result from numerous types of intermolecular interactions. Therefore, we explored the complex molecular relationship in the development of congenital cataracts and provided evidence for the pathogenesis, early diagnosis, and treatment of CC.

Cysteine-rich motor neuron 1 (Crim1) is involved in the function of various organs and affects intercellular connections by regulating integrin signals (Zhang et al. 2016). As a growth-factor-binding protein, crim1 plays an important role in the development of multiple organs, including eye. First, in the lens pit stage, Crim1, which is expressed in the lens epithelial cells and fiber cells, is significantly upregulated and continues to be expressed at a high level throughout embryonic and fetal development. During the maturation of lens fibers in the nucleus, Crim1 is strongly expressed in lens epithelial cells and fiber cells of the lens cortex (Lovicu et al. 2000). In addition, previous studies have shown that Crim1 mutations are responsible for congenital cataracts in subtype and null mutant mouse models (Tam et al. 2018; Pennisi et al. 2007; Chiu et al. 2012; Beleggia et al. 2015). Tight regulation of crim1 activity contributes to the maintenance of lens epithelium, and its deficiency would lead to ectopic differentiation into fiber cells and dramatically alter lens structure (Tam et al. 2018). Lens cells can potentially respond to activin, TGF-β superfamily, and bone morphogenetic protein (BMP) signals, which are related to lens sensing and lens fiber elongation during differentiation. Crim1 interacts with the TGF-β superfamily and BMP. Studies on Xenopus embryos showed that the Crim1 cytoplasmic domain could bind to N-cadherin and β-Catenin, thereby adjusting the makeup of the neurocutaneous adhesion complex. Biochemical analysis of Crim1 showed that Crim1 could bind to BMP and inhibit the production and secretion of BMP (Chiu et al. 2012). In the process of lens development, Crim1 function involves the levels of the β1 integrin. Studies of the β1 integrin signaling pathway revealed that Crim1 can bind to the membrane, thus changing the phosphorylation status of the downstream effectors FAK and ERK and affecting the morphogenesis of the lens (Iyer et al. 2016). Therefore, knowledge on the mechanism of congenital cataracts induced by Crim1 mutations can further predict the occurrence and development of congenital cataracts and provide some guidance for early identification and intervention treatment of congenital cataracts.

Because the human genomes exhibits similarity with the mouse genome, mouse models have been a very useful tool to understand the mechanism of cataracts. We learned much about the pathogenesis of congenital cataracts through a mouse model (Huang et al. 2010; Graw 2009), so we chose the genetic data of mice for analysis. Bioinformatics analysis has become a promising strategy for comprehensive analysis of a large number of data, including complex genetic information. In our study, we used mature bioinformatics tools to screen potential genes in congenital cataracts induced by Crim1 mutations. We chose an open access database called the gene expression omnibus (GEO) database, in which the appropriate mRNA map was selected. The differentially expressed genes (DEGs) between crim1 mutant and wild-type mice were analyzed using the limma package. In our study, we downloaded the GSE62561 mRNA microarray dataset from GEO. The functional and pathway enrichment analysis of DEGs was implemented using the DAVID database. The PPI network was establish through STRING and visualized with Cytoscape. The modularization of the PPI network is obtained through MCODE. This information will lay a foundation for further elucidating the molecular pathway and mechanism of congenital cataracts.

## Materials and Methods

### Subjects and Gene Information

GEO is an international genetic information database consisting of microarrays and next-generation sequencing data that is free to researchers. In this study, a gene expression profile (GSE62561) was searched and screened from the GEO database. As shown in \* MERGEFORMAT Table 1, GSE62561 contained two lens epithelium samples of Crim1 mutant mice and two lens epithelium samples of wild-type mice. The mutant mice is from mice homozygous for a hypomorphic mutation of Crim1 Crim1glcr11 at P60 created by a ENU-induced transition mutation in intron 13. And this variant creates a functional cryptic splice acceptor, resulting in a truncated protein. The wild-type mice that are heterozygous for this mutation is used for controls (Zhang et al. 2016).

**Table 1:** Summary of characteristics of GSE62561

GEO accession	GSE62561
Lens Crim1 glcr11/glcr11	2
Lens Crim1 glcr11/ +	2

### Data Analysis

All data collected from GEO can be processed and normalized in a variety of ways. In this study, two groups of samples were processed and normalized using R language. The DEGs were analyzed by “limma” software package. Comparisons between the two groups were performed under the same experimental conditions. Here, corrected *P* < 0.05 and | logFC | (fold change) > 1 served as the screening criteria.

### GO and KEGG Pathway Analysis

To provide a set of comprehensive functional annotation tools to classify different genes into several groups with different annotations, we used annotation, visualization and enrichment analysis and the DAVID database to analyze the GO and KEGG pathways of the identified DEGs to better understand the biological function of genes and distinguish significantly enriched genes with corrected *P* < 0.05.

### PPI Network Visualization

We developed a quality-controlled pathogenic gene analysis table to evaluate the functional interactions between proteins by simulating the DEG PPI network. The STRING database and Cytoscape were used to visualize the PPI network. Then, molecular module detection (MCODE) is performed to select the appropriate PPI network module. The core genes of the PPI network were detected using CytoHubba.

## Results

### Screening of Differentially Expressed Genes

**Fig. 1:**
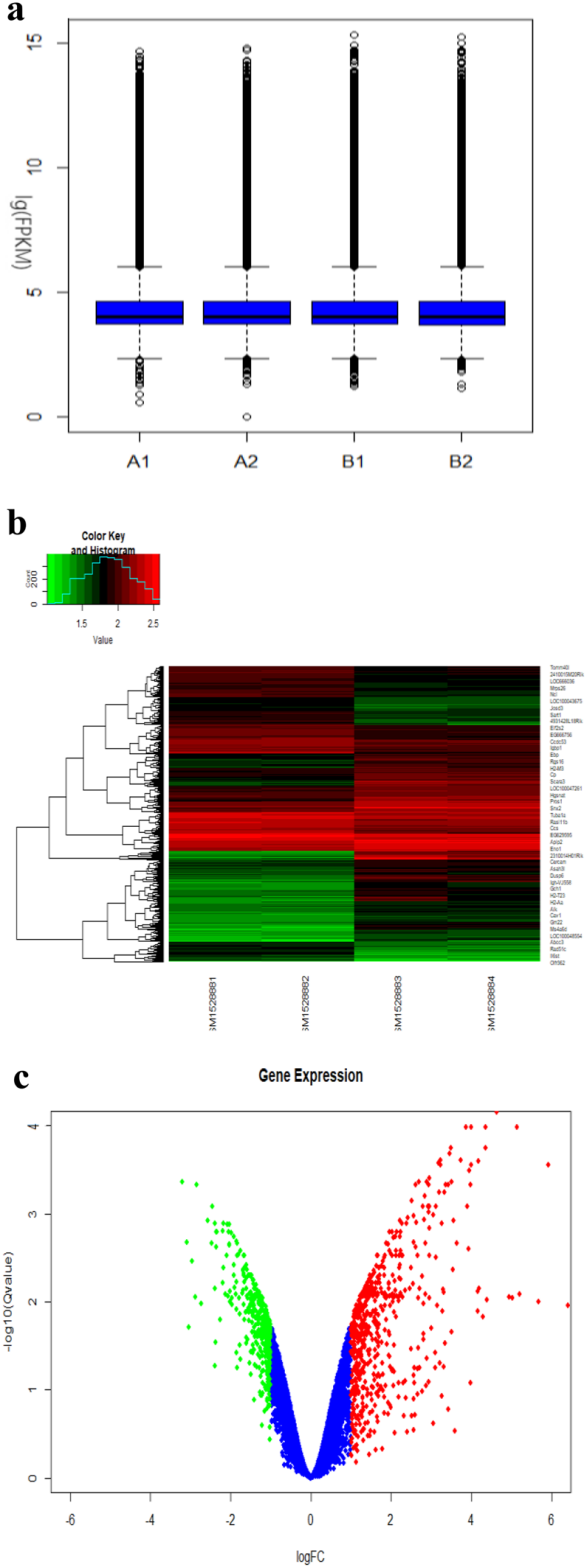
Identification of DEGs in the (CC) dataset (GSE62561) of congenital cataracts induced by Crim mutation. Box diagram of gene expression data after **a** normalization. The horizontal axis represents the sample symbol, and the vertical axis represents the expression value of the gene. The black lines in the block diagram represent the median gene expression (A1 and A2 are wild-type mice, B1 and B2 are Crim mutant mice). **b** Differentially expressed gene heat map (| logFC |> 1&adj. *P*. Val < 0.05). In the heat map, the normalized expression level transitions from green to red. Red indicates overexpression, green indicates low expression, and black indicates normal expression. In addition, each column represents a sample, and each row represents a differentially expressed gene. (Control group: GSM1528881 and GSM1528882. Mutation Group: GSM1528883 and GSM1528884) The volcano map of **c** differentially expressed genes was different. Similar to the heat map, each dot represents a gene. Significantly upregulated genes (adj. *P*. Val < 0.05) are shown in red (fold change > 1), significantly downregulated genes are shown in green (fold change < -1), and normally expressed genes are shown in black (Color figure online)

We found 750 genes altogether in the standardized dataset GSE62561 (Fig. 1a). Basic information about DEGs is in supplement ESM_1. Compared with wild-type mice, 407 genes were upregulated in mutant mouse lens tissue, and 343 genes were downregulated in mutant mouse lens tissue (Fig. 1b, c).

### Enrichment Analysis of GO and KEGG Pathways

**Fig. 2:**
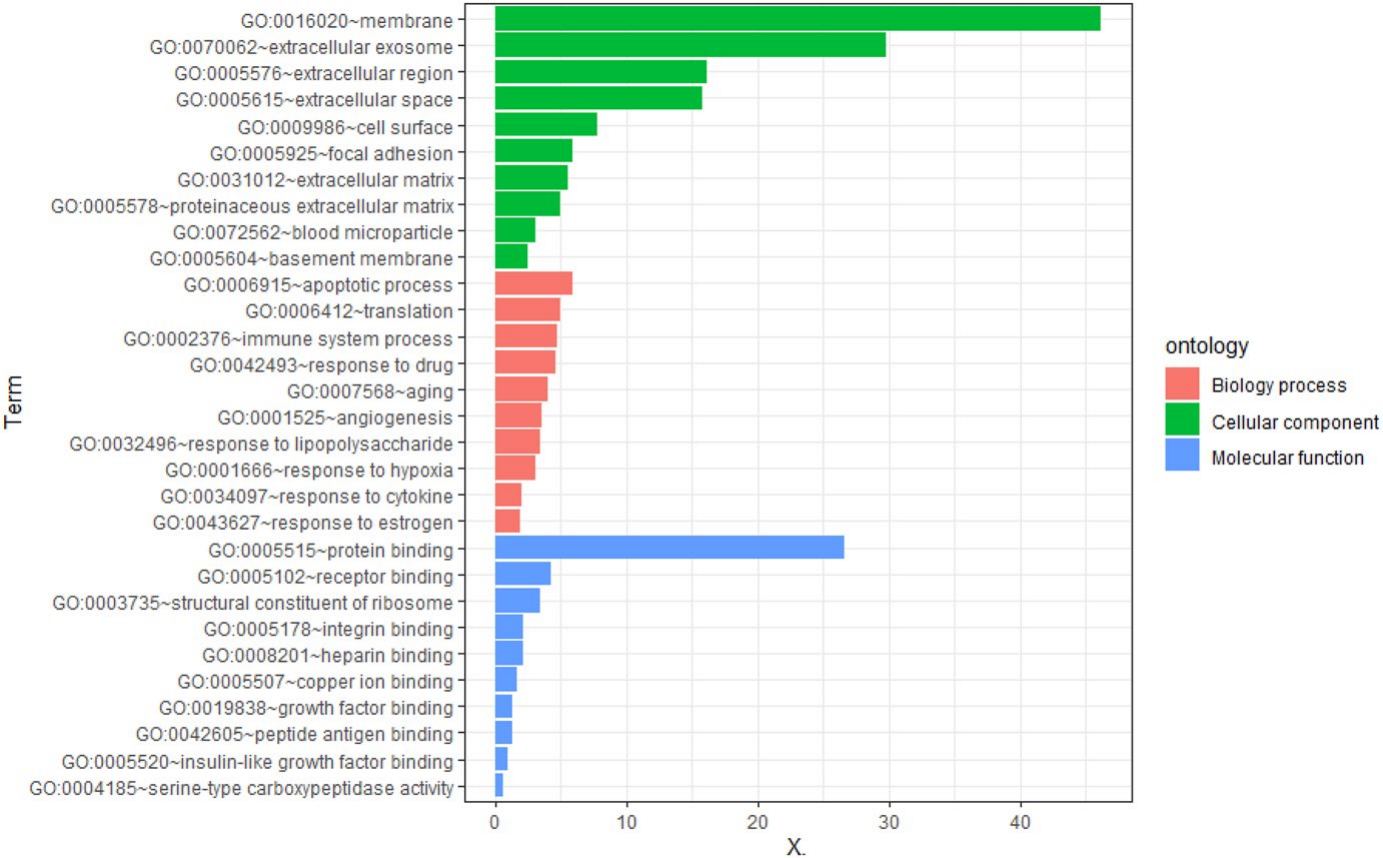
GO analysis of all DEGs annotated bar chart: y-axis represents GO category, including BP (biological process, red), CC (cell component; green), and MF (molecular function; blue); X-axis represents the percentage of differential genes in the total differential genes (Color figure online)

**Fig. 3:**
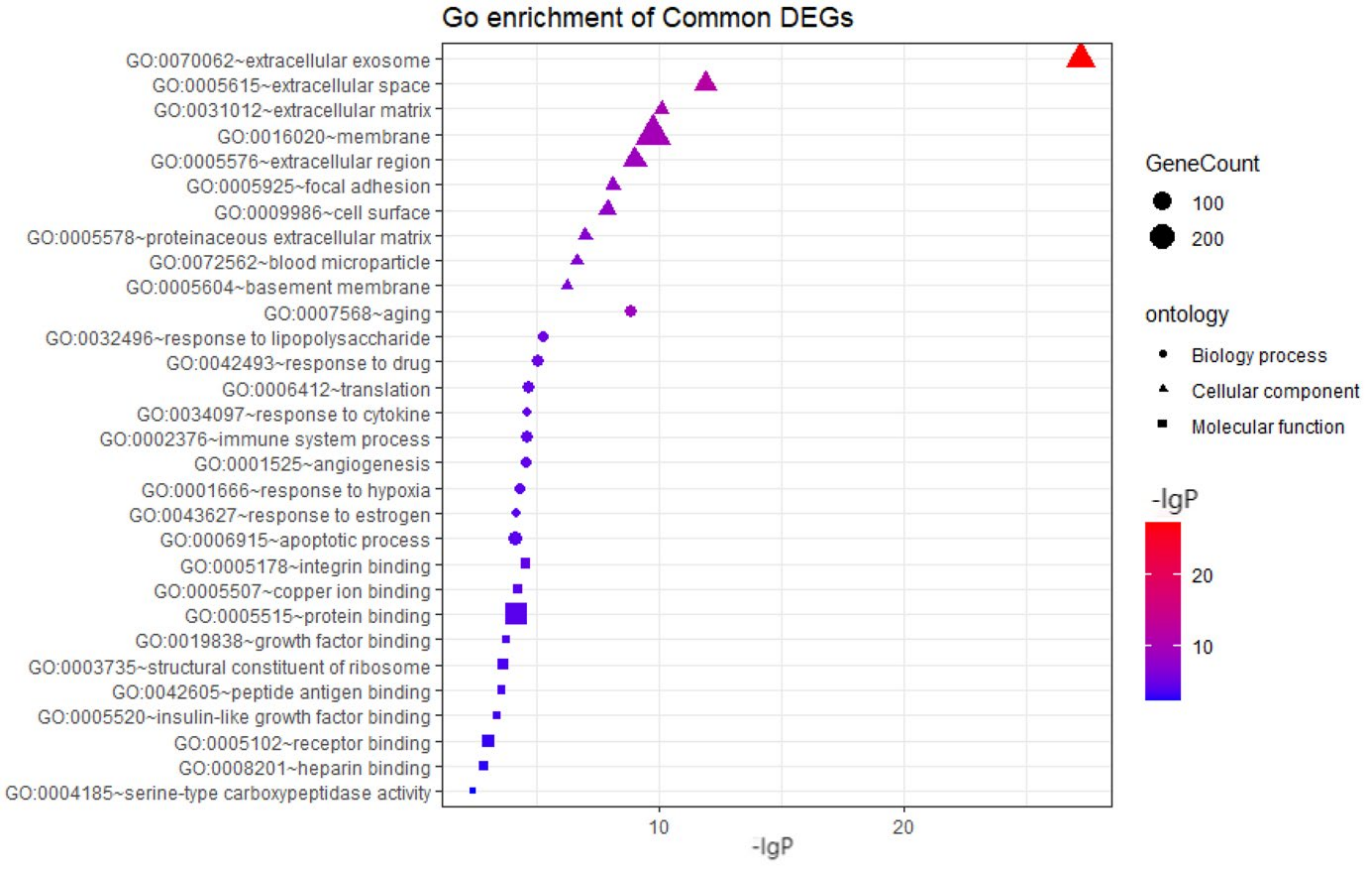
DERNA GO Analysis Notes Bubble Chart: the Y-axis represents the GO category, including BP (bubble shape is round), CC (bubble shape is triangular), and MF (bubble shape is square); the X-axis represents the enrichment fraction. In addition, the color of the bubble also indicates the size of the enrichment fraction (the higher the enrichment fraction, the deeper the red color; the smaller the enrichment fraction, the deeper the blue color). The size of the bubble indicates the number of enriched genes (Color figure online)

**Fig. 4:**
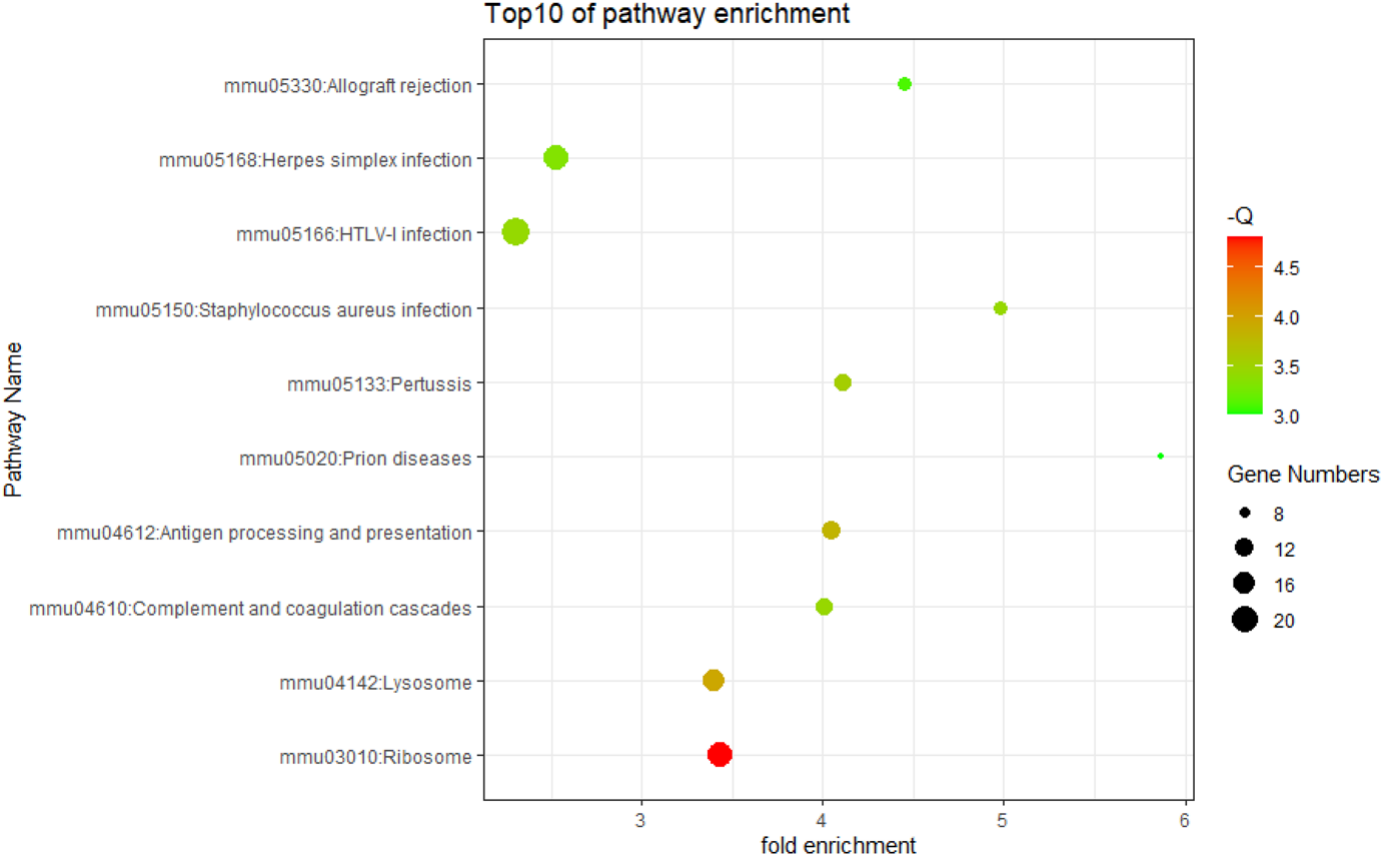
DERNA KEGG functional analysis notes bubble chart: the first 10 significantly enriched KEGG pathways are listed. The Y-axis presents the pathway, and the X-axis indicates the enrichment value. In addition, the color of the bubble also indicates the size of the enrichment fraction (the higher the enrichment fraction, the deeper the red color; the smaller the enrichment fraction, the deeper the blue color). The size of the bubble indicates the number of enriched genes (Color figure online)

The enrichment analysis of GO and KEGG pathways of DEGs was carried out on the DAVID platform for an even better comprehension of the different functions of specific DEGs. GO analysis demonstrated that the DEGs were mainly involved in apoptosis, cell translation, and immune system processes (Fig. 2, 3). KEGG pathway enrichment analysis showed that abundant functions and pathways included ribosome, lysosome, and antigen processing and presentation (Fig. 4).

### PPI Network Construction

**Fig. 5:**
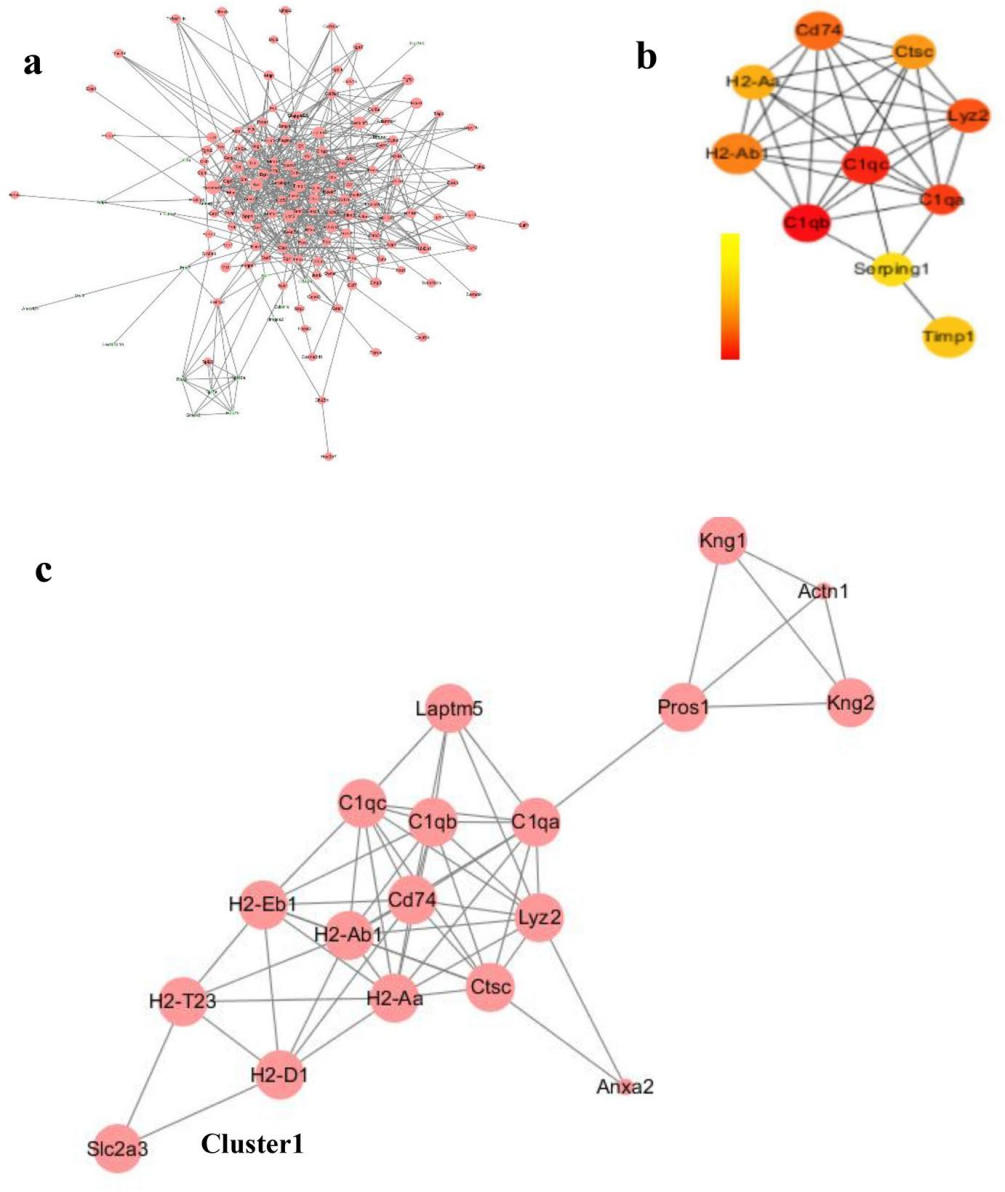
PPI network of DEGs. **a** DE RNAs’ PPI. Green nodes indicate downregulated genes, and red nodes indicate upregulated genes. **b** Screening of the first 10 core genes in the PPI network. Gradual changes in color represent differences in the expression levels of different genes. **c** MCODE plug-in was used to perform module analysis (cluster 1: Score = 6.824 nodes = 18, edges = 58) to join the PPI network of differentially expressed mRNAs.(Parameters: Network Scoring: Include Loops: false Degree Cutoff: 2. Cluster Finding: Node Score Cutoff: 0.2 Haircut: true Fluff: false K-Core: 2 Max. Depth from Seed: 100) (Color figure online)

**Fig. 6:**
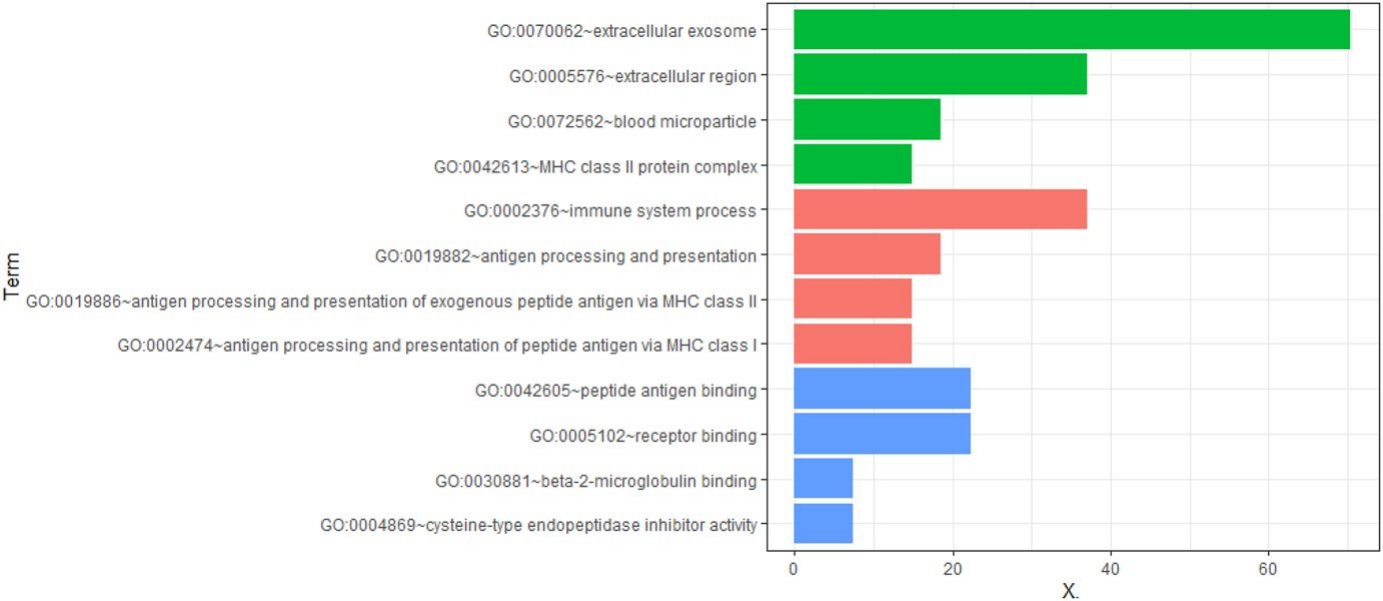
GO analysis notes of functional module cluster 1 bar chart: y-axis indicates GO category, including BF (red), CC (green), and MF (blue); X-axis represents the percentage of differential genes among the total differential genes (Color figure online)

**Fig. 7:**
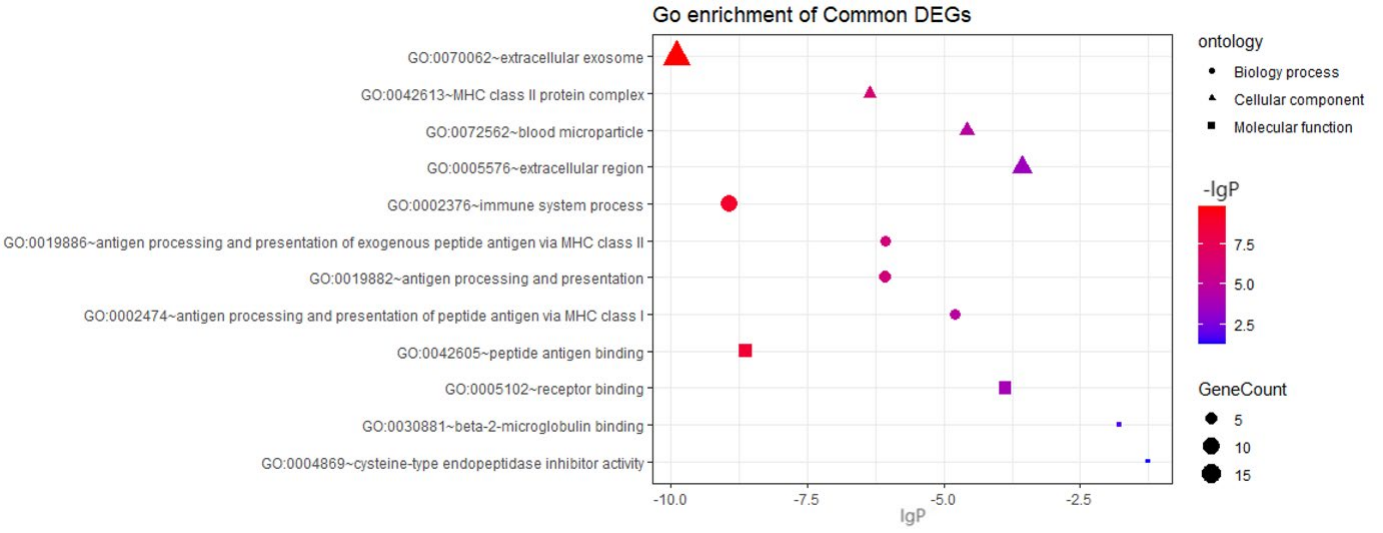
Cluster 1’s GO Analysis Notes Bubble Chart: the Y-axis represents the GO category, including BF (round bubble), CC (triangle), and MF (molecular function; square); X-axis represents the enrichment fraction. In addition, the color of the bubble also indicates the size of the enrichment fraction (the higher the enrichment fraction, the deeper the red color; the smaller the enrichment fraction, the deeper the blue color). The size of the bubble indicates the number of enriched genes (Color figure online)

**Fig. 8:**
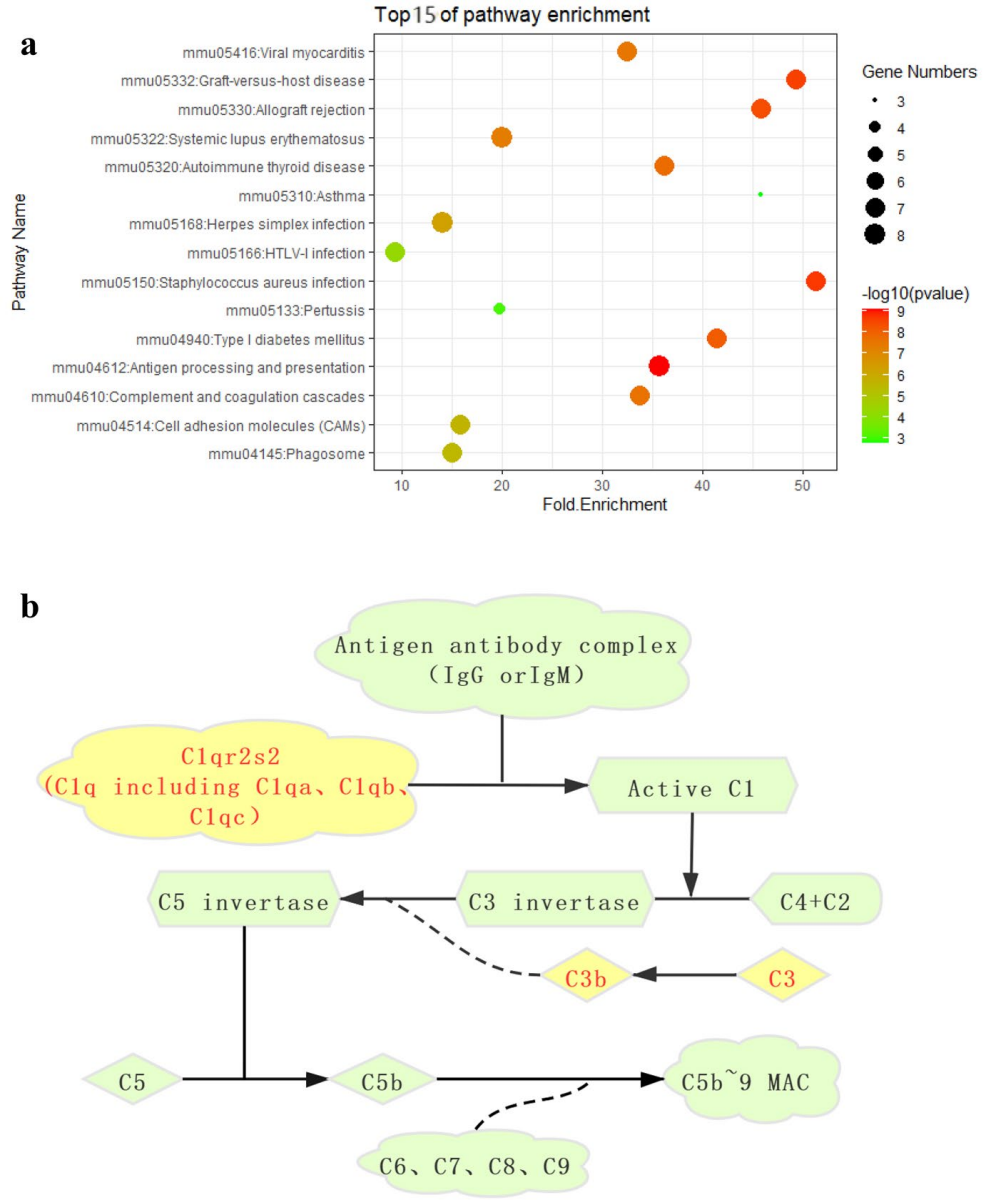
**a** Cluster 1’s KEGG functional analysis notes bubble chart: the first 15 significantly enriched KEGG pathways are listed. The Y-axis indicates the path, and the X-axis indicates the enrichment value. In addition, the color of the bubble also indicates the size of the enrichment fraction (the higher the enrichment fraction, the deeper the red color; the smaller the enrichment fraction, the richer the blue color). The size of the bubble indicates the number of enriched genes. **b** Classical complement activation pathway (Color figure online)

We screened out 202 pathogenic genes from DEGs, excluded unclosely related differentially expressed proteins according to the degree of their interaction, and selected 155 nodes and 566 edges altogether to draw the network of PPIs, which contained 136 genes with excessive expression and 19 genes with reduced expression (Fig. 5a). The top ten core genes of the PPI network were screened by CytoHubba (Fig. 5b), among which C1qa, C1qb, C1qc, and Cd74 are presented in the darkest color and had the most significant effect on the network. Then, using degree ≥ 10 as the cut-off value, a key module consisting of 18 genes (C1qa, C1qb, C1qc, and Cd74) was identified (Fig. 5c). Functional enrichment showed that the genes of this module were primarily related to immune system processes, antigen processing and presentation, MHC class II antigen processing and presentation, and MHC class I antigen processing and presentation (Fig. 6, 7). KEGG pathway enrichment analysis is shown in \* MERGEFORMAT Table 2, and genes in this module were mostly related to *Staphylococcus aureus* infection, graft-versus-host disease and allograft rejection (Fig. 8a). In addition, in order to further explore the role of C1q in congenital cataract, we also explored the classical complement activation pathway (Fig. 8b).

**Table 2:** Function and pathway enrichment analysis of genes in the module

Term	Term description	Count	*P*-value
GO:0070062	Extracellular exosome	19	1.26E-10
GO:0042613	MHC class II protein complex	4	4.48E-07
GO:0072562	Blood microparticle	5	2.67E-05
GO:0005576	Extracellular region	10	2.68E-04
GO:0002376	Immune system process	10	1.18E-09
GO:0019886	Antigen processing and presentation of exogenous Peptide antigen via MHC class II	4	8.41E-07
GO:0019882	Antigen processing and presentation	5	8.58E-07
GO:0002474	Antigen processing and presentation of peptide antigen via MHC class I	4	1.62E-05
GO:0042605	Peptide antigen binding	6	2.31E-09
GO:0005102	Receptor binding	6	1.35E-04
GO:0030881	Beta-2-microglobulin binding	2	0.016276
GO:0004869	Cysteine-type endopeptidase inhibitor activity	2	0.055268
mmu04612	Antigen processing and presentation	8	8.37E-10
mmu05150	*Staphylococcus aureus* infection	7	2.00E-09
mmu05332	Graft-versus-host disease	7	2.56E-09
mmu05330	Allograft rejection	7	4.06E-09

## Discussion

Congenital cataracts often give rise to visual disturbance and even blindness in children worldwide (Li et al. 2020). Early diagnosis is important in postpartum wards or communities given appropriate intervention can lead to a good level of visual function (Roche et al. 2006). In mutant and spontaneous null mouse models, Crim1 is one of the key genes causing congenital cataracts (Tam et al. 2018; Pennisi et al. 2007; Chiu et al. 2012; Beleggia et al. 2015). Therefore, this study will have a certain guiding influence on the early diagnosis and intervention of congenital cataracts by clarifying how Crim1 mutations affect lens development and the molecular mechanism of cataracts in mice through comprehensive bioinformatics analysis. However, the mechanism of the transcriptomic changes induced by deleterized CRIM1 remains to be further investigated, and the analysis and identification of the differential expression of related genes in this paper provide directions for this investigation.

A total of 750 DEGs were identified by downloading the GSE62561 dataset from the GEO website, including 407 genes with excessive expression and 343 genes with reduced expression. GO analysis showed that these differentially expressed genes were primarily affiliated with apoptosis, cell translation, and immune system processes. KEGG pathway enrichment analysis shows that abundant functions and pathways include the processing and presentation of ribosomes, lysosomes, and antigens. In addition, a key module consisting of 18 genes was identified in these DEGs. Among them, C1qa, C1qb, C1qc, and Cd74 exhibited the highest degrees of altered expression. The study of functional enrichment proved that the genes contained in this module were primarily related to immune system processes, antigen processing and presentation, MHC class II antigen processing and presentation, and MHC class I antigen processing and presentation. KEGG pathway enrichment analysis showed that the genes in this module were mostly related to Staphylococcus aureus infection, graft-versus-host disease, and allograft rejection.

### Analysis of C1QA, C1QB, and C1QC

Among the four genes with the highest degree of expression, the C1QA, C1QB, and C1QC genes encode the main component of human complement subfraction C1q (Stasi et al. 2006). In the immune system, C1q is the recognition domain of the initiation protein C1 in the classical complement system that binds to the apoptotic cell membrane or pathogenic agents, triggering the proteolysis cascade of downstream complement proteins that lead to the conditioning and phagocytosis of C3 by macrophages expressing complement receptors (Bialas et al. 2013). As the promoter of the classical complement cascade, C1q is the key modulation node of this pathway. Some studies have shown that the amount of complement C3 in the peripheral blood of sick patients with congenital cataracts is dramatically decreased, and the reduction of complement C3 is a risk factor for congenital cataracts (Shi et al. 2003; Ge et al. 2014; Shao et al. 2017; Li et al. 2017). In addition, complement deposition was also observed in the lens of mice with inflammatory damage (Montalvo et al. 2007). Therefore, the etiological mechanism of congenital cataracts is potentially related to the activation of the complement system. C1q ultimately leads to the conditioning and phagocytosis of C3 by complement receptor macrophages, so the amount of complement C3 is a risk factor for congenital cataracts and indirectly implies the inducing effect of C1q on the occurrence and development of congenital cataracts. In addition, new relationships among TGF-β signaling, the complement system and synaptic removal have been noted. During central nervous system development, the TGF-β cytokine signaling pathway initiates complement-dependent synaptic refinement by regulating C1q expression (Town et al. 2008; Fonseca et al. 2004; Afagh et al. 1996; Stevens et al. 2007; Bialas et al. 2013). Abnormal upregulation of complement may trigger synaptic elimination pathways in neurodevelopment and lead to synaptic loss in the disease. Synaptic loss and/or dysfunction represent the primary characteristics of degenerative neurodegeneration (Bialas et al. 2013). In this study, the C1qa, C1qb, and C1qc genes were upregulated in mutant mice, suggesting that the early development of congenital cataracts may activate the classical complement system, leading to synaptic disorders and neurodegenerative diseases, thus promoting the development of CC. C1q exhibits decreased expression in the normal major central nervous system; however, in the glaucoma mouse model, C1q is upregulated and synaptically relocated in the adult retina in the preliminary periods of the disease (Stevens et al. 2007). In addition, some researchers have found that the pathogenesis of congenital cataracts caused by TDRD7 deficiency may be related to the immune response, defence response, and related genes LY86, C1QA, C1QB, and C1QC (Shao et al. 2015). Therefore, C1qa, C1qb, and C1qc may also be involved in the pathogenesis of congenital cataracts caused by Crim1 mutation, and these proteins may also be related to neurodegenerative diseases (Fig. 8b).However, the complement C1q protein domain was significantly downregulated in the lens of Tdrd7 cataractous mutant mice, which we hypothesized might be related to mutations in different genes. However, in the lenses of Tdrd7 cataract mutant mice, the complement C1q protein domain was significantly downregulated, contrary to our findings, which we speculate may be related to mutations in different genes. At present, the specific mechanism of lens transcriptomic changes in Tdrd7 cataract mutant mice is not clear, and more research is needed in the future.

### Analysis of CD74

As a type II transmembrane protein, CD74 (also known as MHC class II invariant chain) is affiliated with the transport of MHC class II molecules in antigen presenting cells. CD74 is a member of the MIF (macrophage migration inhibitory factor) receptor complex and its signal components CD44 and/or chemokine receptors CXCR2 and CXCR4 (Schwartz et al. 2012; Leung et al. 2004; Benedek et al. 2013). MIF can aggravate allergic and inflammatory diseases in humans (Rossi et al. 1998; Nagata et al. 2015). In addition, as a widely expressed pluripotent cytokine, MIF exhibits extensive immune and inflammatory activities. The cell surface morphology of CD74 facilitates binding with MIF to activate its related functions. Its high expression is related to different diseases with the same inflammatory characteristics (Sanchez-niño et al. 2009; Martín-ventura et al. 2009; Marsh et al. 2009; Borghese et al. 2011). The combination of MIF and CD74 mediates ERK activation and cell proliferation and inhibits apoptosis and prostaglandin E2 production (Calandra et al. 2003; Wang et al. 2014). In the lens, MIF mRNA is expressed in differentiated epithelial cells during development, which may enhance the growth of cells and cytodifferentiation (Wang et al. 2017). The increase in MIF mRNA abundance in the lenses of cataract rats is affiliated with the proliferation of undifferentiated epithelial cells (Wen et al. 1996). In addition, blocking the binding and downstream signal transduction of cytokines/chemokines, MIF and downstream signal transduction on monocytes and macrophages through CD74 receptors can inhibit brain reactive T cells without causing systemic immunosuppression (Wang et al. 2017; Vandenbark et al. 2013). Therefore, the upregulation of Cd74 may suggest that the occurrence and development of congenital cataracts caused by Crim1 mutations is related to the immune system, and pathway analysis also shows that Cd74 is affiliated with the immune system process, antigen processing and presentation, MHC class II antigen processing and presentation and MHC class I antigen processing and presentation.

### Limitations

This paper lays a foundation for further elucidation of the molecular pathways and mechanisms of congenital cataracts. However, this article also has some limitations. First, the occurrence of cataracts in Crim1glcr11 mice may be associated with transcriptomic changes. At the same time, these changes are more likely to be the result of cataract formation, as Crim1glcr11 mice used for transcriptomic analysis have cataracts at P60. However, the etiological link between transcriptome changes and cataracts has not been clarified here, and further experiments are needed to verify this in the future. In addition, the expression of these key genes and their changes at the transcriptomic level will need to be further verified in future experiments.

## Conclusion

In conclusion, through comprehensive biological analysis of the GSE62561 gene expression profile, a total of 750 DEGs were identified, of which C1qa, C1qb, C1qc, and Cd74 may be related to the occurrence and development of congenital cataracts caused by Crim1 mutations. Further studies are needed to clarify the association. The progression of congenital cataracts may be induced and aggravated by the immune system, classical complement system or antigen presentation pathway. Pathway intervention may have a certain guiding significance for the treatment of congenital cataracts. However, the predictive values of these genes and their pathways require further study before use in clinical practice. The underlying mechanism of the activity of these genes needs to be clarified, which will help to outline the predictive network of these genes.

## Supplementary Information

Below is the link to the electronic supplementary material.

Supplementary file1 (XLSX 65 KB)

## Data Availability

The data used to support the findings of this study are included within the article.
